# Influence of long-term fertilization on soil microbial biomass, dehydrogenase activity, and bacterial and fungal community structure in a brown soil of northeast China

**DOI:** 10.1007/s13213-014-0889-9

**Published:** 2014-04-22

**Authors:** Peiyu Luo, Xiaori Han, Yan Wang, Mei Han, Hui Shi, Ning Liu, Hongzhi Bai

**Affiliations:** 1 Present Address: College of Land and Environment, Shenyang Agricultural University, Dongling Road 120, Shenyang, 110866 Liaoning China; 2 National Engineering Laboratory for Efficient Utilization of Soil and Fertilizer Resources, Dongling Road 120, Shenyang, 110866 Liaoning China; 3 Foreign Language Teaching Department, Shenyang Agricultural University, Dongling Road 120, Shenyang, 110866 Liaoning China

**Keywords:** Long-term fertilization, Bacterial and fungal community structure, Soil microbial biomass carbon, Dehydrogenase activity, PCR-DGGE

## Abstract

In this study, the effect of mineral fertilizer and organic manure were evaluated on soil microbial biomass, dehydrogenase activity, bacterial and fungal community structure in a long-term (33 years) field experiment. Except for the mineral nitrogen fertilizer (N) treatment, long-term fertilization greatly increased soil microbial biomass carbon (SMBC) and dehydrogenase activity. Organic manure had a significantly greater impact on SMBC and dehydrogenase activity, compared with mineral fertilizers. Bacterial and fungal community structure was analyzed by polymerase chain reaction (PCR)-denaturing gradient gel electrophoresis (DGGE). Long-term fertilization increased bacterial and fungal ribotype diversity. Total soil nitrogen (TN) and phosphorus (TP), soil organic carbon (SOC) and available phosphorus (AP) had a similar level of influence on bacterial ribotypes while TN, SOC and AP had a larger influence than alkali-hydrolyzable nitrogen (AHN) on fungal ribotypes. Our results suggested that long-term P-deficiency fertilization can significantly decrease soil microbial biomass, dehydrogenase activity and bacterial diversity. N-fertilizer and SOC have an important influence on bacterial and fungal communities.

**Electronic supplementary material:**

The online version of this article (doi:10.1007/s13213-014-0889-9) contains supplementary material, which is available to authorized users.

## Introduction

Soil microorganisms are important to agroecosystems. They are involved in key roles, such as soil aggregate formation, soil humus formation, nutrient cycling, decomposition of various compounds and other transformations (Lynch and Bragg 1985; Zak et al. 1994; Wu et al. 2011). Fertilization is the most common management of agricultural soils. Organic and inorganic fertilizers are primarily used to increase crop yield, and in short-term fertilizer experiments, they have no significant effect on microbial community (Crecchio et al. 2001; Marschner et al. 2001); however, in long-term fertilizer experiments, they can affect the function, community structure, and population of soil microorganisms (Marschner et al. 2003; Cinnadurai et al. 2013). Fertilization usually strongly favors the accumulation of bacterial residues (Joergensen et al. 2010; Murugan and Kumar 2013) and increases soil microbial biomass (Peacock et al. 2001; Parham et al. 2002; Kaur et al. 2005; Ebhin Masto et al. 2006).

In the long term, repeated fertilization may result in shifts in the functionality and quality of soils by directly or indirectly changing the soil's physical, chemical, and biological properties as it changes available nutrient level and fertility. Some microorganisms may proliferate, and others may be suppressed. Microorganisms appear to be very sensitive to management practices such as mineral fertilizer and manure addition (Vries et al. 2006; Walsh et al. 2012). To understand the relationship between microorganisms and soil nutrients is a precondition to be certain how microorganisms affect soil nutrient cycling.

The brown soils are some of the most primary arable soils; however, there are few reports on the influence of long-term fertilization on soil microbial biomass, dehydrogenase activity, bacterial and fungal community structure under a rotation cropping system in a brown soil. Long-term studies are precious tools to understand changes of soil productivity and quality (Mitchell et al. 1991). In 1979, a long-term research site was established in Liaoning Province of northeast China, to evaluate the effects of fertilization practices on soil ecosystem processes and nutrient dynamics under a maize-maize-soybean rotation. This study was conducted to test the long-term impact of fertilization on soil microbial biomass, dehydrogenase activity, bacterial and fungal community structure, using chemical analysis, molecular fingerprinting and exploratory multivariate analysis. The results of this study may provide more insight into the effects of fertilization on soil bacterial and fungal communities, as well as subsequent effects on nutrient cycling.

## Materials and methods

### Experimental site and investigation design

The study was conducted in the semi-humid region of Shenhe district, Shenyang (40°48′ N and 123°33′ E) of Liaoning Province, China. The long-term fertility experiments were begun in 1979. Basic chemical properties of experimental soil in 1979 are detailed in Table S1. The mean annual temperature and precipitation was 7.0–8.1 °C and 574–684 mm, respectively. Soil in the study site was clay brown loam (48 % sand, 29 % silt and 23 % clay at 0–20 cm depth) and falls under the class of Alfisols with hydromica as a dominant clay mineral. Eight treatments (four replicates each) were established in 1979 using a randomized block design in 32 plots (10 × 5 m) under a rotation of maize-maize-soybean: no fertilizer (C), N (mineral nitrogen fertilizer), NP (mineral nitrogen and phosphate fertilizer), NPK (mineral nitrogen, phosphate and potassic fertilizer), pig manure (M), MN (pig manure and mineral nitrogen fertilizer), MNP (pig manure, mineral nitrogen and phosphate fertilizer), MNPK (pig manure, mineral nitrogen, phosphate and potassic fertilizer). The mineral fertilizers were applied in the form of urea, calcium superphosphate and potassium sulphate, and the application rates are detailed in Table S2. Tillage (20 cm depth) in general was done either the day before or on the day of planting. Weeding in the crop field was done by handpicking.

### Soil sampling

Soil samples were taken from the top 0 to 20 cm soil from the crop rotation system during April 2012 (maize year). The 0–20 cm layer of the soil was sampled with 5 cm diameter cores between the row and the fertilizer band. A first set of nine cores were taken from different locations (randomly) in each plot and pooled in plastic bags and used for analysis. All samples were placed in an ice box until arrival in the laboratory and stored at 4 °C. The soil samples for DNA analysis were stored at −20 °C until analysis. The other set was sieved through 2 mm and divided into two sub-samples. One was weighed and oven-dried at 105 °C to constant weight, and the other was air-dried and finely ground for chemical analysis. The dried sample was weighed, and gravimetric soil water content was calculated by difference and expressed as a percentage on a dry soil weight basis (Topp 1993).

### Soil chemical analysis

Soil pH was determined with a glass electrode using a soil/water ratio of 1:2.5. Soil organic carbon (SOC) and total nitrogen (TN) were determined by dichromate oxidization (Mebius 1960) and Kjeldahl digestion (Bremner 1965) respectively. Total soil phosphorus (TP) and potassium (TK) were digested by HF-HClO_4_ (Jackson 1958) and determined by molybdenum-blue colorimetry and flame photometry, respectively. Available phosphorus (AP) and potassium (AK) in the soil were extracted by sodium bicarbonate (Olsen et al. 1954) and ammonium acetate (Carson 1980), respectively. Alkali-hydrolyzable nitrogen (AHN) in the soil was determined as described by Xiong et al. (2008). Soil microbial biomass carbon (SMBC) was determined by a chloroform fumigation-extraction method (Vance et al. 1987). Dehydrogenase activity was determined by the reduction of triphenyltetrazolium chloride to triphenylformazan as described by Casida et al. (1964).

### DNA extraction and PCR amplification

Total genomic DNA was extracted from 250 mg of soil using the PowerSoil™ DNA Isolation Kit (MoBio Laboratories, Inc., USA). Amplification reactions were performed as described by Beauregard et al. (2010).

### DGGE analysis

PCR products were analyzed using a DCODE Universal Mutation Detection System (Bio-Rad Laboratories, Hercules, CA, USA) with 16 × 18 cm glass plates and 1 mm spacers. The different DGGE conditions corresponding to fungi and bacteria are described in Table S3. Gels were stained with SYBR Gold (Invitrogen, USA) diluted 1:10000, visualized on a UV transilluminator, and photographed (GelDoc, Bio-Rad Laboratories, USA). The bands that migrated to different positions were considered to be different ribotypes.

### Recovery of DNA from DGGE gel

DNA was recovered from DGGE gel using the method of Sheng et al. (2013). The sequencing reactions were performed with a DNA sequencing kit, BigDye™ Terminator v3.1 (Applied Biosystems, Foster City, CA, USA), and the reaction products were analyzed with an ABI 3730xl DNA Analyzer (Applied Biosystems, USA). Partial 16S rRNA and 18S rRNA sequences were identified based on similarity using the National Centre for Biotechnology Information (NCBI) online standard BLAST (Basic Local Alignment Search Tool) program (http://www.ncbi.nlm.nih.gov/).

### Statistical analysis

The Shannon-Weaver diversity index (Shannon 1948) was calculated from the number of bacterial and fungal bands detected on DGGE to compare the ribotype diversity between different fertilization treatments using Quantity One. Cluster analysis was performed using Quantity One by UPGMA. Canonical correspondence analysis was performed on the presence-absence matrix of DGGE banding patterns, and redundancy analysis using CANOCO 4.5 to evaluate the relationships between the DGGE patterns of different samples and the possible influence of fertilization treatments. The data were subjected to analysis of variance, using IBM SPSS Statistics 19.0 for Windows (IBM, Inc., Armonk, NY, USA). A probability level of 5 % was adopted for accepting or rejecting null hypotheses. Tukey’s honestly significant difference test for all-pairwise comparisons were calculated after ANOVA to compare treatment means.

## Results

### Soil chemical properties

Long-term fertilization resulted in significant differences in soil chemical properties (Table 1). Compared with organic fertilized soils, mineral fertilizer treatments showed a significant lower in pH value. Fertilization significantly increased the stocks of TN, AP and AHN. SOC and AK in organic fertilizer treatments were significantly higher than that in mineral fertilizer treatments. Fertilization significantly increased the amount of TP as compared to N treatment.

**Table 1:** Chemical properties of long-term fertilizer treatments

Treatment	pH (H_2_O)	TN (g kg^−1^)	SOC (g kg^−1^)	TP (g kg^−1^)	TK (g kg^−1^)	AP (mg kg^−1^)	AK (mg kg^−1^)	AHN (mg kg^−1^)
NP	5.32 ± 0.07 d	1.60 ± 0.01 d	11.04 ± 0.08 f	0.51 ± 0.005 c	13.50 ± 0.07 f	31.34 ± 0.50 e	85.09 ± 1.17 f	138.40 ± 2.51 e
NPK	5.17 ± 0.13 e	1.74 ± 0.06 c	10.96 ± 0.15 f	0.53 ± 0.002 c	13.34 ± 0.10 g	29.49 ± 1.16 e	88.48 ± 2.03 e	151.37 ± 2.17 d
N	5.30 ± 0.10 d	1.63 ± 0.03 d	11.47 ± 0.17 e	0.30 ± 0.003 d	13.73 ± 0.09 e	2.28 ± 0.30 f	86.44 ± 1.02 ef	137.09 ± 5.26 e
C	5.95 ± 0.13 c	1.55 ± 0.03 e	9.93 ± 0.06 g	0.31 ± 0.005 d	13.39 ± 0.03 fg	0.90 ± 0.11 f	96.59 ± 1.07 d	113.29 ± 2.27 f
MNP	6.23 ± 0.08 ab	1.86 ± 0.03 b	16.19 ± 0.16 c	0.87 ± 0.037 a	14.51 ± 0.09 b	172.16 ± 6.08 a	122.99 ± 2.11 b	193.73 ± 0.90 a
MNPK	6.12 ± 0.12 b	1.97 ± 0.02 a	17.63 ± 0.10 a	0.85 ± 0.031 a	14.79 ± 0.05 a	162.94 ± 2.97 b	137.21 ± 1.01 a	163.15 ± 3.05 c
MN	6.23 ± 0.07 ab	1.92 ± 0.03 ab	14.98 ± 0.02 d	0.76 ± 0.027 b	14.08 ± 0.04 c	140.80 ± 2.18 c	105.74 ± 2.68 c	179.09 ± 8.41 b
M	6.42 ± 0.09 a	1.99 ± 0.09 a	16.45 ± 0.13 b	0.54 ± 0.001 c	13.86 ± 0.10 d	126.97 ± 1.14 d	103.03 ± 2.11 c	149.23 ± 0.36 d

The mean value ± standard deviation (*n* = 4)

*TN* total N, *SOC* soil organic C, *TP* total P, *TK* total K, *AP* available P, *AK* available K, *AHN* alkali-hydrolyzable N

### Soil microbial biomass C and dehydrogenase activity

**Fig. 1:**
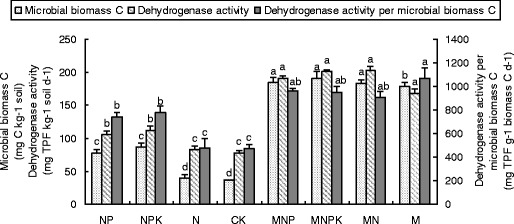
Effect of different treatments on soil microbial biomass C, dehydrogenase activity and dehydrogenase activity per microbial biomass C under long-term application of mineral fertilizer and organic manure. *C* no fertilizer, *NP* mineral NP fertilizer, *NPK* mineral NPK fertilizer, *N* mineral N fertilizer, *M* pig manure, *MNP* pig manure plus mineral NP fertilizer, *MNPK* pig manure plus mineral NPK fertilizer, *MN* pig manure plus mineral N fertilizer. *Vertical bars* are standard deviation; not followed by the same *letter* indicate significant difference (*P* < 0.05)

Fertilization significantly increased SMBC and dehydrogenase activity after long-term application. Compared with mineral fertilizers, organic manures had significantly greater impact on SMBC and dehydrogenase activity. The variation trend of dehydrogenase activity per SMBC (Fig. 1) was similar to that of SMBC and dehydrogenase activity. Compared with P-sufficiency fertilization, P-deficiency fertilization significantly decreased soil microbial biomass and dehydrogenase activity.

### Microbial diversity

**Fig. 2:**
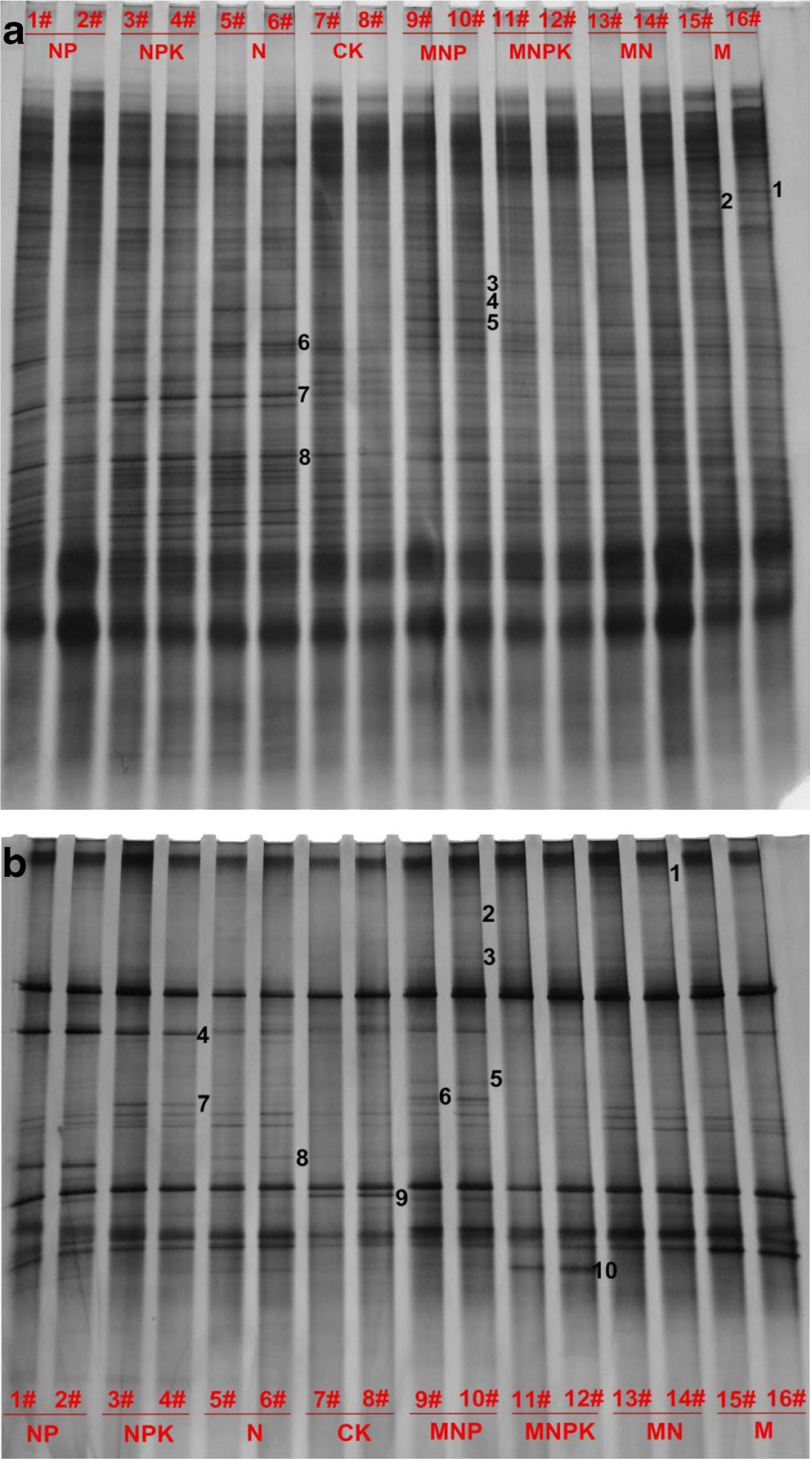
Denaturing gradient gel electrophoresis (DGGE) banding patterns of **a** bacterial 16S rRNA gene and **b** fungal 18S rRNA gene retrieved from different soil samples

Long-term fertilization significantly increased bacterial and fungal diversity (Table 2). Fertilization treatments increased the Shannon-Weaver diversity index of bacteria and fungi, and increased soil microbial taxonomic richness, i.e., the number of different bands detected in samples. DGGE revealed the presence of 33–38 bands (NP 36,37; NPK 38,37; N 34,33) in mineral treatments and 40–44 bands in organic treatments (MNP 44,43; MNPK 44,43; MN 40,41; M 42,42) corresponding to bacteria, while there were only 28 and 29 bands in the C treatment (Fig. 2a). DGGE revealed the presence of 21–23 bands (NP 22,23; NPK 23,21; N 22,23) in mineral treatments, and 21–27 bands in organic treatments (MNP 27,27; MNPK 22,23; MN 24,24;M 22,21), corresponding to fungi, while there were only 15 and 17 bands in the C treatment (Fig. 2b). P-deficiency fertilization significantly decreased bacterial but not fungal diversity, compared with P-sufficiency fertilization. Long-term fertilization increased operational taxonomic units (OTUs) related to the below microorganisms, *Acidobacteria* bacterium, gamma proteobacterium, *Mortierella*, *Knufia petricola* and *Zygomycetes* in mineral treatments, while *Firmicutes* bacterium, *Cyanobacterium*, *Myxococcales* bacterium, *Acidobacteria* bacterium, beta proteobacterium, *Mortierella*, *Aleuria aurantia* and *Chytridiomycota* in organic manure treatments. Fertilization suppressed OTUs related to *Nectria mariannaeae* (Fig. 2a, b, Tables 3 and 4).

**Table 2:** Diversity of fungal and bacterial communities under long-term application of mineral fertilizer and organic manure

Treatment	Shannon-Weaver diversity index^a^	
	Bacteria	Fungi
NP	3.536	2.769
NPK	3.574	2.713
N	3.420	2.756
C	3.303	2.574
MNP	3.612	2.951
MNPK	3.635	2.761
MN	3.545	2.805
M	3.521	2.785

^a^Shannon-Weaver index, H ′ = − ∑ *p*_*i*_ ln *p*_*i*_, p_i_ represents that of the specific band brightness accounts for the proportion of all bands brightness in one lane (Shannon 1948)

**Table 3:** BLAST (Basic Local Alignment Search Tool) results for bacterial sequences found in soil samples

Specific bands	Most related isolate from GenBank (% sequence similarity by BLAST^a^)	GenBank accession no.
Band 1	Uncultured *Firmicutes* bacterium clone GASP-KA1S3_F05 16S ribosomal RNA gene, partial sequence (98 %)	EU297149.1
Band 2	Uncultured *Cyanobacterium* clone GASP-MA2S2_E12 16S ribosomal RNA gene, partial sequence (98 %)	EF662970.1
Band 3	Uncultured *Myxococcales* bacterium clone Plot18-E05 16S ribosomal RNA gene, partial sequence (99 %)	FJ889278.1
Band 4	Uncultured *Acidobacteria* bacterium clone S2-046 16S ribosomal RNA gene, partial sequence (99 %)	KF182982.1
Band 5	Uncultured beta proteobacterium partial 16S rRNA gene, clone pLA12 (100 %)	HF934065.1
Band 6	Uncultured gamma proteobacterium gene for 16S rRNA, partial sequence, isolate: sd-jx75 (100 %)	AB690765.1
Band 7	Uncultured *Acidobacteria* bacterium clone Acido.wet.ACETH07 16S ribosomal RNA gene, partial sequence (100 %)	GU374498.1
Band 8	Uncultured *Acidobacteriaceae* bacterium clone CS6 16S ribosomal RNA gene, partial sequence (99 %)	JQ771962.1

^a^97 % sequence similarity is the minimum requirement for identity to the species level (Stackebrandt and Goebel 1994)

**Table 4:** BLAST (Basic Local Alignment Search Tool) results for fungal sequences found in soil samples

Specific bands	Most related isolate from GenBank (% sequence similarity by BLAST)	GenBank accession no.
Band 1	*Aleuria aurantia* 18S ribosomal RNA gene, partial sequence (97 %)	DQ248951.1
Band 2	Uncultured *Chytridiomycota* clone T5P1AeD07 18S ribosomal RNA gene, partial sequence (99 %)	GQ995425.1
Band 3	*Mortierella indohii* strain Mort-300 18S ribosomal RNA gene, partial sequence (98 %)	EU688965.1
Band 4	*Knufia petricola* strain CBS 726.95 18S ribosomal RNA gene, partial sequence (99 %)	KC978739.1
Band 5	Uncultured *Mortierella* isolate DGGE gel band K19b 18S ribosomal RNA gene, partial sequence (99 %)	JX560305.1
Band 6	*Mortierella indohii* strain Mort-300 18S ribosomal RNA gene, partial sequence (100 %)	EU688965.1
Band 7	*Zygomycetes* sp. AM-2008a isolate AF010 18S ribosomal RNA gene, partial sequence (99 %)	EU428774.1
Band 8	Uncultured *Mortierella* isolate DGGE gel band K19b 18S ribosomal RNA gene, partial sequence (99 %)	JX560305.1
Band 9	*Nectria mariannaeae* genes for 18S ribosomal RNA (99 %)	AB099509.1
Band 10	Uncultured fungus clone T3_IV_3a_15 18S ribosomal RNA gene, partial sequence	EF628887.1

### Cluster analysis of DGGE fingerprints

**Fig. 3:**
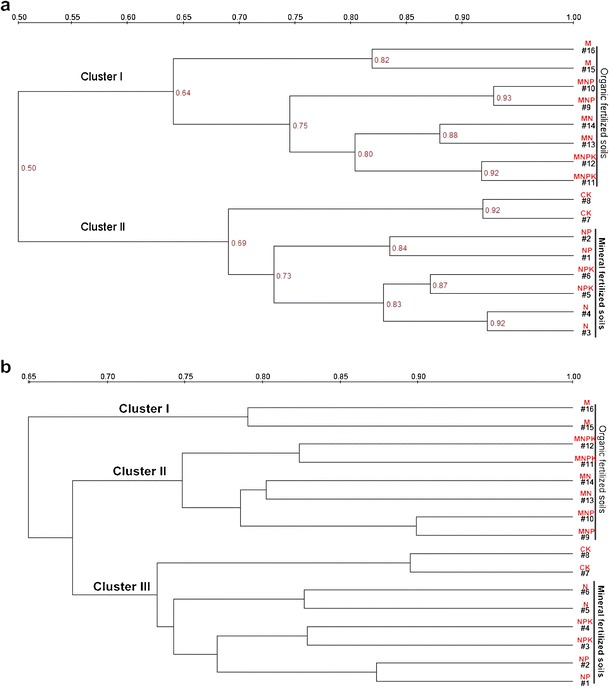
Cluster analysis of denaturing gradient gel electrophoresis (DGGE) banding patterns of **a** bacterial 16S rRNA gene and **b** fungal 18S rRNA gene retrieved from soil samples

Analysis of the four replicates of each treatment showed good reproducibility of DGGE banding patterns (data not shown); therefore, only two replicates were shown in the DGGE profiles. Duplicates of the DGGE banding patterns of 16S rRNA genes and 18S rRNA genes of different soil samples are shown in Fig. 2a and b. The DGGE band position, number, and density had high similarity between the duplicates for all soil samples, which suggested that the duplicate DNA analysis was good enough for distinguishing the difference between the bacterial community and fungal community among the eight treatments in this study. DGGE banding patterns revealed that most DGGE bands were very similar, which inferred that the bacterial (Fig. 2a) and fungal (Fig. 2b) species with those bands were stable and commonly existed in brown soil. The observed differences of banding patterns were likely owing to several bands or band density. Cluster analysis of DGGE banding patterns of bacterial communities showed that the dendrogram was divided into Clusters I and II. Cluster I consisted of all soil samples obtained from organic fertilizer treatments, and Cluster II contained the soil samples collected in mineral fertilizer treatments as compared to control (C) (Fig. 3a). Cluster analysis of DGGE banding patterns of fungal communities showed that the dendrogram was divided into Clusters I, II and III. Cluster I contained the soil samples obtained from manure (M) treatment, Cluster II consisted of the soil samples obtained from treatments of manure applied with mineral fertilizers, and Cluster III contained the soil samples collected in mineral fertilizer treatments and C treatment (Fig. 3b). The results showed significant differences between organic fertilized soils and mineral fertilized soils; organic manure had a particular role in soil microbial structure.

### Effect of soil chemical properties on soil microbial communities

**Fig. 4:**
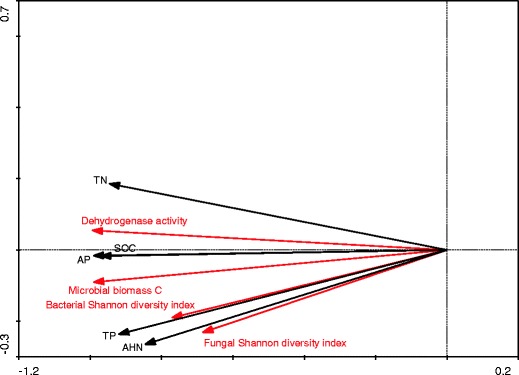
Redundancy analysis biplot depicting the relationship among soil properties, dehydrogenase activity, microbial biomass C, fungal and bacterial Shannon diversity index. *TN* total N, *SOC* soil organic C, *TP* total P, *AP* available P, *AHN* alkali-hydrolyzable N

**Fig. 5:**
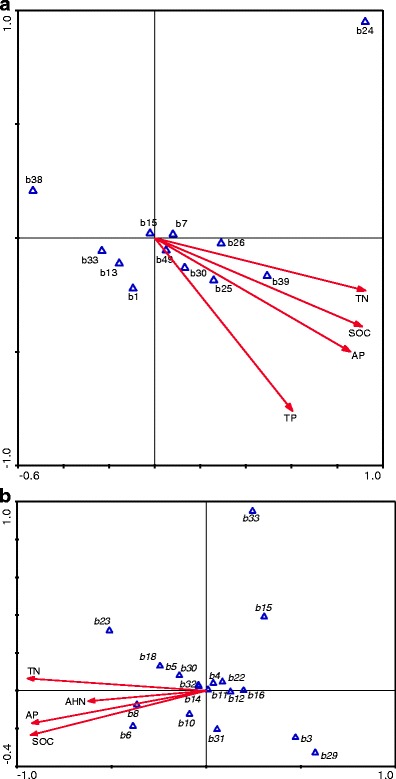
CCA biplot depicting the relationship between **a** bacterial and **b** fungal ribotypes with regard to selected soil properties. Selected soil properties effects are represented by vectors. Ribotypes are represented by *triangles* and named according to the migration position on the gels

Redundancy analysis showed the relationship among selected soil chemical properties, SMBC and dehydrogenase activity. Fungal and bacterial Shannon diversity index was significant, the coordinate from the first two ordination axes explained 98 % of the variance, and the significance (Monte Carlo permutation tests) of all canonical axes was *P* = 0.03 (Fig. 4). SMBC, dehydrogenase activity, fungal and bacterial Shannon diversity index were positively correlated with TP, TN, SOC, AP and AHN. CCA revealed substantial alteration of bacterial (Fig. 5a; *P* = 0.05) and fungal (Fig. 5b; *P* = 0.05) communities with regard to selected soil chemical properties. TN, TP, SOC and AP had a similar level of influence on bacterial ribotypes while TN, SOC and AP had a larger influence than AHN on fungal ribotypes.

## Discussion

### Soil chemical properties

The application of mineral fertilizer significantly decreased the soil pH. Such a decrease in pH values in long-term fertilization might be due to the nitrification of NH_4_^+^ which produces H^+^ ions thus increasing soil acidity (Birkhofer et al. 2008). On the other hand, pH values in organic manure treatments were higher than that in mineral fertilizer treatments, which might be due to enrichment of cations (Murugan and Kumar 2013).

Application of organic manure increased stocks of SOC and SMBC. On one hand, this could be caused by the higher amount of organic carbon and microbial biomass carbon added by organic manures, and on the other hand, increased carbon input resulted in long-term nutrient accumulation (Kaur et al. 2005; Joergensen et al. 2010; Wang et al. 2010), so TN, TP and TK in organic manure treatments were significantly higher than those in mineral fertilizer treatments. Long-term mineral fertilizer applications resulted in loss of organic matter (Jenkinson 1991; Aref and Wander 1997). The greater losses in simple than in balanced fertilizer treatments were most likely because of crop residue, as higher yields, and hence greater organic matter inputs in the form of crop residue, in balanced treatments. Organic manure significantly increased AP, which may be due to the release of P from manure (7.4 g total P kg^−1^ manure).

### Soil microbial biomass and dehydrogenase activity

Soil microbial biomass has been commonly defined as an indicator of soil fertility and quality, which is easily influenced by soil management (Pankhurst et al. 1995). The SMBC content ranged from 77.5 mg kg^−1^ (C treatment) to 203.2 mg kg^−1^ in the MNPK plots (Fig. 1). Except for the N treatment, SMBC contents of all fertilized treatments were significantly higher than the C treatment. These results indicated that long-term deficiency of P significantly affected microbial biomass in soil as compared to P-sufficiency fertilization. SMBC content was thus increased as a result of fertilizer applications, corroborating previous observations (Kanazawa et al. 1988; Goyal et al. 1992). In our study, 0.7–1.2 % of SOC was present as SMBC. The percentage of total C present as SMBC usually ranges from 0.27 % to over 7.0 %, depending on soil type, vegetation cover, management and analytical methods (Anderson and Domsch 1989). SMBC and dehydrogenase activity were significantly correlated to SOC and TN in loess (Chu et al. 2007). In this study, in addition to SOC and TN, SMBC and dehydrogenase activity were also positively correlated with TP, AP and AHN (Fig. 4). This demonstrated that factors influencing microbial biomass in different types of soil were different.

The metabolic activity of microbial community can be evaluated by determining ratios of intracellular enzyme activities with respect to SMBC (Deng et al. 2006). Except for the N treatment, dehydrogenase activity per SMBC in fertilizer treatments was significantly higher than that in the C treatment (Fig. 1), indicating that balanced fertilization resulted in higher microbial metabolic activity than nutrient-deficiency fertilization (Chu et al. 2007).

### Influence of fertilization on soil microbial community

It is commonly known that each DGGE band represents at least one ribotype (Muyzer et al. 1993); therefore, in this study, fertilizer treatments, especially organic manure treatments, significantly increased diversity of bacterial and fungal ribotypes. The change of bacterial community structure by organic fertilizer had been observed in several long-term field experiments (Marschner et al. 2003; Sun et al. 2004). In our study, organic fertilizer divided bacterial communities into two clusters (Fig. 3a) and fungal communities (Fig. 3b) into three clusters. This might be due to direct influence of the microorganism in the manure or promoting effect of the manure on the growth of particular indigenous microorganism in soils after long-term application of the manure.

Soil fertility can influence the biomass, activities, and diversity of the soil microbial community in different ways (Fox and MacDonald 2003; Liu et al. 2008; Wei et al. 2008). Fertilization could directly or indirectly cause changes in the soil's physical, chemical and biological properties. Fertilization could surely affect microbial growth and competitiveness as different groups of microorganisms could vary in their ability to process the various nutrient forms found in soil. In this study, TP, TN, SOC, AP and AHN affected diversity of bacteria and fungi (Fig. 4). In particular, we showed that the availability of nutrients such as C, N, and P can influence soil microbial growth and activity (Silva and Nahas 2002; Broeckling et al. 2008).

The PCR-DGGE method is commonly used to detect the most abundant genotypes in soil microbial communities and/or other environments, as it is known to show sequence variants forming more than 1 % of the target sequences (Muyzer et al. 1993). However, if compared to the multitude of microorganisms that could be found in soil, a relatively low number of ribotypes were detected. It was reported that different land management practices produced a selective influence on microbial community diversity (Hagn et al. 2003; Wu et al. 2008). In our study, different fertilization during 33 years had a profound effect on the soil microbial community. Changes at a fine taxonomic scale were revealed by the PCR–DGGE analysis of whole fungal and bacterial rRNA gene fragments in soil extracts, which indicated that TN, TP, SOC and AP had a similar level of influence on bacterial ribotypes (Fig. 5a), while TN, SOC and AP had a larger influence than AHN on fungal ribotypes (Fig. 5b). Some specific ribotypes were found mostly in fertilized soils suggesting favorable conditions for the proliferation and activity of specific microbial taxa (Beauregard et al. 2010). In our study, the dominating fungal and bacterial taxa were different in the mineral and organic fertilized soils. Additionally, some OTUs related to certain microorganisms were just found in mineral fertilizer treatments (*Knufia petricola* and *Zygomycetes*) or organic manure treatments (*Firmicutes* bacterium, *Cyanobacterium*, *Myxococcales* bacterium, *Aleuria aurantia* and *Chytridiomycota*) (Fig. 2a, b, Tables 3 and 4). In the long term, such changes might result in shifts the quality and functionality of soils. Some microorganisms might be proliferated, and others might be suppressed. Differences in the microbial community structure in our study might be due to the previously mentioned reasons. On the other hand, this might be due to differences in root exudation. Root exudation represents an important source of soil carbon for microorganisms and it was influenced by plant nutrient status (Singh and Pandey 2003).

This study demonstrated that the type of fertilization could significantly change the structure of soil microbial communities most likely by changing the soil chemical properties and its fertility status. Except for the P-deficient treatment, fertilizer treatments especially organic manure treatments significantly increased SMBC and dehydrogenase activity. Fertilization increased bacterial and fungal diversity. Our results suggest that phosphorus fertilizer influences the composition of soil bacterial and fungal communities. TN, TP, SOC and AP had a similar level of influence on bacterial ribotypes while TN, SOC and AP had a larger influence than AHN on fungal ribotypes. Our results also suggest that nitrogen fertilizer and SOC have important influence on soil bacterial and fungal communities. Thus our future study should focus on functional microorganisms which are closely related with soil N and P nutrient.

## Electronic supplementary material

Below is the link to the electronic supplementary material.

Table S1 (DOC 26 kb)

Table S2 (DOC 34 kb)

Table S3 (DOC 40 kb)
