# Supplementary material for: Influence of long-term fertilization on soil microbial biomass, dehydrogenase activity, and bacterial and fungal community structure in a brown soil of northeast China
Source: Ann Microbiol. 2014 Apr 22;65(1):533–42. doi: 10.1007/s13213-014-0889-9 (PMC4331610; doi:10.1007/s13213-014-0889-9)
Supplement: Supplementary file 1 — (DOC 26 kb) [file 13213_2014_889_MOESM1_ESM.doc]

**Table S1 Basic chemical properties of experimental soil in 1979**

|  | Soil pH (H_2_O) | TN  (g kg^-1^) | SOC (g kg^-1^) | TP  (g kg^-1^) | TK  (g kg^-1^) | AP  (mg kg^-1^) | AK  (mg kg^-1^) | AHN  (mg kg^-1^) |
| --- | --- | --- | --- | --- | --- | --- | --- | --- |
| 1979 | 6.50 | 0.80 | 15.90 | 0.38 | 20.10 | 6.50 | 97.9 | 105.5 |
