# Supplementary material for: Influence of long-term fertilization on soil microbial biomass, dehydrogenase activity, and bacterial and fungal community structure in a brown soil of northeast China
Source: Ann Microbiol. 2014 Apr 22;65(1):533–42. doi: 10.1007/s13213-014-0889-9 (PMC4331610; doi:10.1007/s13213-014-0889-9)
Supplement: Supplementary file 2 — (DOC 34 kb) [file 13213_2014_889_MOESM2_ESM.doc]

**Table S2** **Application rates of long-term fertilizer treatments a**

| Treatment | Application rates of Mineral fertilizer (kg ha-1) | | | Application rates of organic manure (t ha-1) |
| --- | --- | --- | --- | --- |
|  | N c | P2O5 | K2O |  |
| NP | 120/30 b | 60/90 | 0/0 | 0/0 |
| NPK | 120/30 | 60/90 | 30/60 | 0/0 |
| N | 120/30 | 0/0 | 0/0 | 0/0 |
| C | 0/0 | 0/0 | 0/0 | 0/0 |
| MNP | 120/30 | 60/90 | 0/0 | 27.0/0 |
| MNPK | 120/30 | 60/90 | 30/60 | 27.0/0 |
| MN | 120/30 | 0/0 | 0/0 | 27.0/0 |
| M | 0/0 | 0/0 | 0/0 | 27.0/0 |

a The crop planted:1979-1980 maize,1981 soybean; repeat every three years

b Application rates of maize year/ Application rates of soybean year

c urea, calcium superphosphate and potassium sulphate were converted to effective components (N, P2O5­­, K2O)
