# Supplementary material for: Influence of long-term fertilization on soil microbial biomass, dehydrogenase activity, and bacterial and fungal community structure in a brown soil of northeast China
Source: Ann Microbiol. 2014 Apr 22;65(1):533–42. doi: 10.1007/s13213-014-0889-9 (PMC4331610; doi:10.1007/s13213-014-0889-9)
Supplement: Supplementary file 3 — (DOC 40 kb) [file 13213_2014_889_MOESM3_ESM.doc]

**Table S3** PCR-DGGE conditions used in this study

| Microbial  group | Primers (5'-3') ^a^ | PCR conditions | DGGE conditions |
| --- | --- | --- | --- |
| Bacteria | pA | 3 min at 94 °C , 35 cycles of 30 s at 94 °C , 30 s at 58 °C , 1 min at 72 °C , and final extension for 5 min at 72 °C | 8 % (w/v) polyacrylamide gels with a denaturant gradient of 45–70 % (100 % is defined as 40 % formamide and 7 M urea) at 60 °C. 60 V for 16 h |
|  | AGAGTTTGATCCTGGCTCAG |  |  |
|  | pH |  |  |
|  | AAGGAGGTGATCCAGCCGCA |  |  |
|  | 341F-GC (GC clamp ) | 2 cycles of 1 min at 94 °C , 45 s at 65 °C , 1 min at 72 °C ; decreased annealing temperature by 1 °C every 2 cycles until 55 °C, final extension for 5 min at 72 °C |  |
|  | TACGGGAGGCAGCAG |  |  |
|  | 534R |  |  |
|  | ATTACCGCGGCTGCTGG |  |  |
| Fungi | FR1-GC (GC clamp) | 3 min at 95 °C , then 35 cycles of 30 s at 95 °C , 45 s 50 °C , 1 min at 72 °C , and final extension for 10 min at 72 °C | 7 % (w/v) polyacrylamide gels with a denaturant gradient of 40–60 % at 60 °C. 100 V for 20 min, then 60 V for an additional 16 h |
|  | AICCATTCAATCGGTAIT |  |  |
|  | FF390 |  |  |
|  | CGATAACGAACGAGACCT |  |  |

^a^ For bacteria ,primer pairs used in the first round of nested PCR are presented first
